# Stay in the Game: Comprehensive Approaches to Decrease the Risk of Sports Injuries

**DOI:** 10.7759/cureus.76461

**Published:** 2024-12-27

**Authors:** Florian Forelli, Ayrton Moiroux-Sahraoui, Mathias Nielsen-Le Roux, Nicholas Miraglia, Maxime Gaspar, Maria Stergiou, Andreas Bjerregaard, Jean Mazeas, Maurice Douryang

**Affiliations:** 1 Sports Rehabilitation, Orthosport Center, Domont, FRA; 2 Orthopedic Surgery, Clinic of Domont, Domont, FRA; 3 Physical Therapy, Miraglia Private Clinic, Trieste, ITA; 4 Rehabilitation, Centre National du Football (CNF) Clairefontaine, Clairefontaine-en-Yvelines, FRA; 5 Sports Medicine, Universidad Europea de Madrid (UEM) Real Madrid, Madrid, ESP; 6 Physiotherapy, University of Southern Denmark, Odense, DNK; 7 Physiotherapy and Physical Medicine, University of Dschang, Dschang, CMR

**Keywords:** biomechanical assessment, injury prevention strategies, nutrition, psychological resilience, sports injuries

## Abstract

Recurrent sports injuries present complex challenges that extend beyond the playing field, impacting athletes' physical well-being, mental resilience, and financial stability. This review outlines a comprehensive framework designed to prevent and manage these setbacks, empowering athletes to achieve sustained performance and recovery. This multidimensional issue requires an integrative approach encompassing physical rehabilitation, psychological resilience, and nutritional strategies. Physically, injury prevention relies on tailored rehabilitation programs that address athletes' specific injury histories and sport-related demands. Techniques such as biomechanical assessments and neuromuscular training correct faulty movement patterns, while workload monitoring ensures a balance between training and recovery. Strength and conditioning programs have demonstrated success in reducing injury rates and enhancing musculoskeletal resilience. Psychologically, reinjury anxiety and mental disengagement are key barriers to recovery and performance. Interventions such as goal setting, mindfulness, and cognitive restructuring rebuild confidence and foster readiness to return to play. Addressing psychological well-being is as critical as physical health in promoting sustained performance. Nutrition is equally pivotal, with balanced macronutrient intake supporting energy and tissue repair and micronutrients (magnesium, manganese, vitamin D, selenium, zinc, coenzyme Q10, etc.) bolstering bone health. Anti-inflammatory nutrients and proper hydration reduce muscle strain and aid recovery, while personalized dietary strategies meet the specific demands of individual sports. By integrating these physical, psychological, and nutritional dimensions, this framework empowers athletes, coaches, and medical teams to minimize injury recurrence and enhance long-term performance, ensuring athletes remain competitive and resilient in their sporting pursuits.

## Introduction and background

In the competitive realm of sports, where success often hinges on physical endurance and peak performance, athletes frequently encounter the risk of injuries. While injury is a well-acknowledged aspect of any athletic career, the recurrence of injuries introduces a unique set of challenges that extend beyond the physical [1,2]. Recurrent sports injuries can sideline athletes for prolonged periods, threaten the longevity of their careers, and pose substantial obstacles to reaching their full potential [3]. Furthermore, the ramifications of recurrent injuries are wide-ranging, affecting mental and financial aspects of an athlete's well-being [4,5]. Repeated injuries can lead to chronic physical limitations, decreased mobility, and increased risk of long-term conditions such as osteoarthritis and persistent pain [6]. Additionally, the mental toll from battling recurrent injuries may manifest in psychological challenges, including anxiety, depression, and diminished self-confidence, which further complicate the path to recovery [7]. Financially, recurrent injuries are costly, not only due to medical treatments but also due to lost earnings and the potential for shortened careers [8].

This narrative review delves into a multidimensional evidence-based support, multi-faceted approach to minimizing the risk of recurrent injuries. By focusing on physical rehabilitation, psychological resilience, and preventive strategies, our aim is to provide insights that empower athletes, coaches, and medical professionals to foster an environment where peak performance is sustained and injuries are kept at bay. A comprehensive understanding of these strategies can equip athletes to remain competitive, resilient, and primed to "stay in the game."

## Review

Understanding the impact of injuries

Injury prediction and risk factor identification studies are performed using a variety of study designs and a multitude of statistics. While some authors have highlighted statistical significance and clinical relevance in their findings, careful consideration of methodological design is essential to accurately assess the generalizability and practical implications of these results. Recurrent injuries can have significant physiological, psychological, and economic consequences for athletes. Studies have shown that athletes who experience recurrent injuries face elevated risks of long-term health issues and are at a higher risk of long-term physical issues, such as chronic pain and decreased mobility [9-11]. Moreover, the psychological toll of dealing with repeated injuries can lead to issues like depression, anxiety, and decreased self-esteem [12-15]. Financial implications are similarly substantial, with recurrent injuries leading to increased treatment costs, lost game time, and career implications [16]. Injury prevention is crucial for both amateur and professional athletes, though the risks and consequences can differ significantly. For recreational athletes, addressing the impact of a sedentary lifestyle is crucial, as repetitive injuries can discourage continued participation in sports. This may result in prolonged inactivity, leading to reduced cardiovascular fitness, muscle atrophy, weight gain, and an increased risk of metabolic disorders such as diabetes or hypertension. [17]. Meanwhile, for professional athletes, recurring injuries not only risk ending careers prematurely but also pose a serious threat to performance levels, potentially impacting long-term success and stability in their field [18]. Prioritizing prevention strategies can help mitigate these risks across all levels of sport [19,20]. To identify risk factors for injuries, a four-step sequence has been suggested by van Mechelen et al. It involves (1) establishing the extent of the injury or problem, (2) establishing the etiology and mechanisms of the injury, (3) introducing a preventive measure, and (4) assessing the effectiveness by repeating step 1 [21]. To prevent injury recurrences, it is crucial to identify the risk factors associated with them. Research indicates that previous injuries are the most significant risk factor for future injuries. Hägglund et al. found and demonstrated that previous hamstring injuries in soccer players significantly increased their risk of subsequent hamstring injuries [20]. However, previous injuries may not be isolated risk factors but indicators of underlying biomechanical or physiological imbalances. Perhaps it should be noted that previous injuries as a risk factor could be the result of other unidentified risk factors. Therefore, it is important to delve deeper than simply stating he/she has been injured before. Other risk factors include inadequate rehabilitation, premature return to play, improper biomechanics, and potentially also a misdiagnosis [1]. Inadequate rehabilitation is often due to inadequate objectives for the completion of the rehabilitation process, lack of follow-up assessment, or incomplete healing of the initial injury [22]. Returning to play too early can exacerbate an injury, leading to further damage and a longer recovery period. Improper biomechanics, such as poor running or jumping techniques, can place excessive strain on certain body parts, increasing the likelihood of reinjury [1]. Rehabilitation is essential in preventing recurrent injuries. This involves not only treating the injury but also addressing the underlying causes and ensuring the athlete is fully recovered before returning to play. Rehabilitation should be tailored to the individual needs of the athlete, considering the specific demands of their sport and their injury history. Fousekis et al. emphasized the importance of individualized rehabilitation programs [23]. They found that personalized rehabilitation programs, which included strength training, flexibility exercises, and sport-specific drills, were more effective in preventing recurrent injuries compared to generic programs. Additionally, incorporating neuromuscular training and strength training which focuses on improving coordination and control has been shown to reduce the risk of reinjury [24,25]. Injury prevention must also include a focus on athletic training, helping the athlete gradually return to the specific physical demands of their sport. This process can be guided by frameworks like Taberner et al.'s "Control to Chaos" continuum, which provides a structured progression from controlled rehabilitation exercises to the unpredictable dynamics of full competition [26]. Such an approach ensures that athletes are not only healed but also adequately prepared to handle the stresses of their sport, reducing the risk of reinjury.

Mechanisms of injury and multifactorial considerations

The mechanism of sports injuries is inherently multifactorial, involving the interplay of intrinsic and extrinsic factors that contribute to an athlete's susceptibility to harm. A comprehensive understanding of these mechanisms requires recognizing how biological, biomechanical, environmental, and psychological components interact dynamically to create conditions for injury (Table 1).

**Table 1:** Key factors and mechanisms underlying sports injuries

Factor type	Examples	Relevance to injury
Intrinsic factors	Biomechanical deficiencies (e.g., anterior pelvic tilt), muscle strength, flexibility, joint stability, age, sex, previous injury history	These factors predispose athletes to injuries through inherent vulnerabilities in their anatomy or physiology
Extrinsic factors	Training volume, surface type, environmental variables, equipment quality (e.g., inadequate footwear)	External conditions exacerbate risks, often interacting with intrinsic vulnerabilities to increase injury likelihood
Dynamic interaction of factors	Fatigue impairing neuromuscular control, leading to altered joint mechanics and increased risk of ligament injuries	Illustrates how isolated disruptions can cascade into broader injury risks through system-wide interactions
Load and fatigue	Overtraining without recovery, cumulative workload exceeding adaptive capacity, impaired proprioception due to fatigue	Core contributors to both acute and overuse injuries, with fatigue leading to compensatory movements that overload tissues
Psychological influences	Fear of reinjury, anxiety, altered movement patterns, cautious gait post-rehabilitation	Psychological factors can modify physical performance and movement patterns, increasing injury susceptibility

Dynamic Interaction of Factors

Injury rarely results from a single cause; instead, it emerges from the cumulative impact of multiple interacting variables. Bittencourt and colleagues emphasize that injuries are products of complex systems, where even minor disruptions in one factor, such as fatigue, can cascade through the system to amplify injury risk [27]. For example, muscular fatigue may impair neuromuscular control, altering joint mechanics and increasing the likelihood of ligamentous injuries during high-intensity activities [28]. This cascading effect highlights how interconnected factors synergistically contribute to an elevated risk, challenging the perception that these elements act independently.

**Figure 1:**
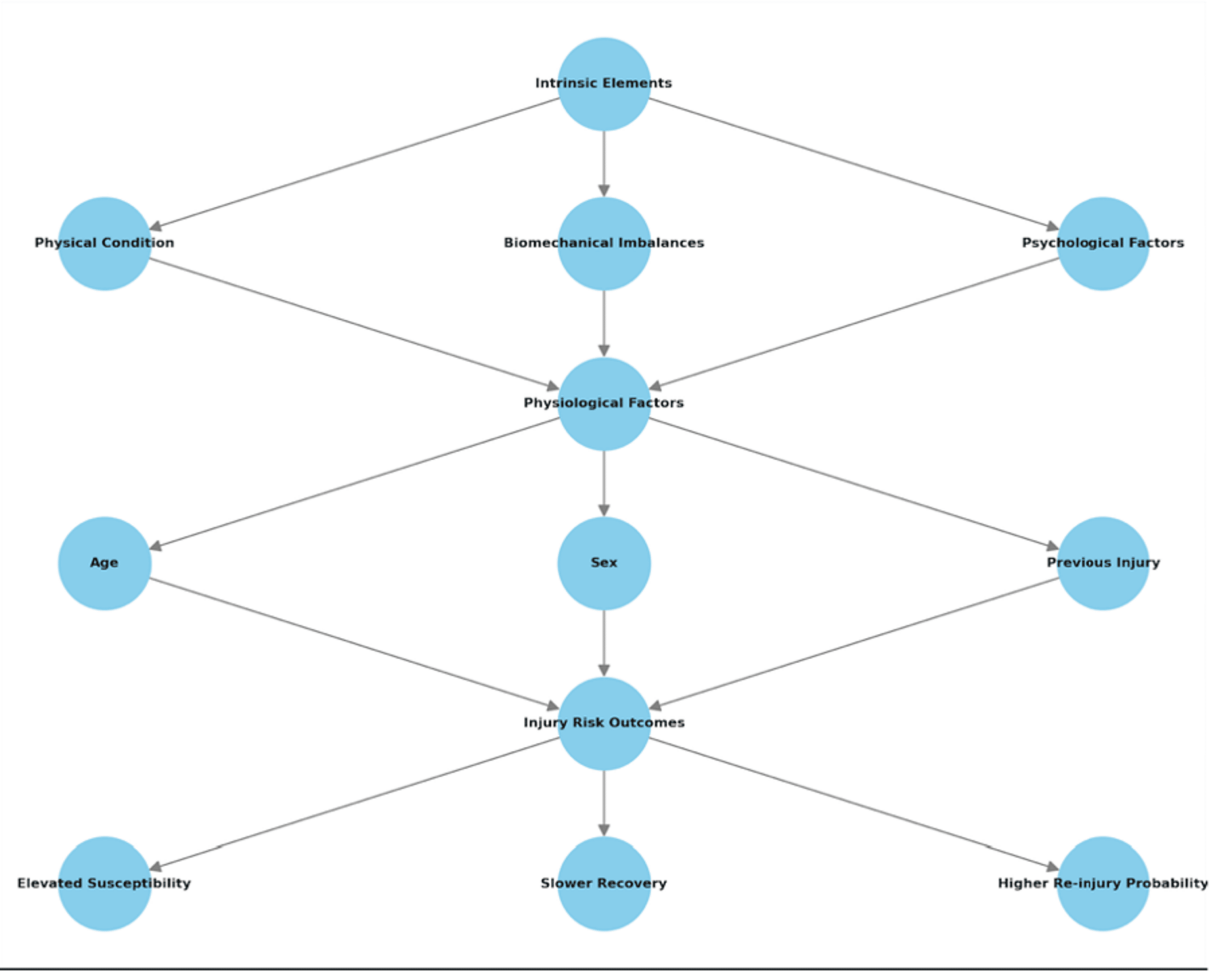
Interaction of intrinsic and physiological factors leading to injury risk

Intrinsic factors pertain to an athlete's individual characteristics, including anatomical alignment, muscle strength, flexibility, and joint stability. For instance, biomechanical deficiencies such as an anterior pelvic tilt or excessive pronation during running can place abnormal stress on muscles and joints, predisposing athletes to injuries like hamstring strains or stress fractures (Table 2) [29-31]. These intrinsic elements frequently interact with physiological factors, including age, biological sex, and a history of previous injuries, widely recognized as one of the strongest predictors of future injury. These interactions create a complex network of risk factors that collectively influence injury susceptibility and recovery potential (Figure 1) [20,32].

**Table 2:** Biomechanical deficiencies and associated injuries ACL: anterior cruciate ligament

Biomechanical deficiency	Description	Examples of associated injuries
Anterior pelvic tilt	Increased forward tilt of the pelvis, often due to weak core and tight hip flexors	Hamstring strains, lower back pain
Excessive pronation during running	Over-rotation of the foot inward during gait, causing misalignment of the lower limb	Plantar fasciitis, shin splints, stress fractures
Scapular dyskinesis	Abnormal movement or positioning of the shoulder blade during arm motion	Rotator cuff tears, shoulder impingement syndrome
Knee valgus (knock knees)	Inward collapse of the knees during activities such as landing or squatting	ACL injuries, patellofemoral pain syndrome
Ankle instability	Recurrent giving way or instability of the ankle joint, often due to prior sprains or ligament laxity	Ankle sprains, chronic instability, peroneal tendinopathy
Insufficient hip external rotation	Reduced ability of the hip joint to rotate outward, limiting range of motion and stability	Labral tears, hip impingement syndrome, stress fractures

Extrinsic contributors include external conditions such as training volume, surface type, and environmental variables. Sudden increases in workload, such as progressing too quickly in intensity or duration, are well-documented as key risk factors for overuse injuries [28]. Additionally, poor equipment, such as inadequate footwear, can exacerbate biomechanical issues, leading to strain on specific body regions. Studies show that mismatches between physical preparation and the demands of competition are common triggers for acute injuries, such as ligament sprains during rapid deceleration or cutting movements [33-36].

The Role of Load and Fatigue

Training load and fatigue play central roles in the injury mechanism, bridging intrinsic and extrinsic factors. When the cumulative workload exceeds an athlete's capacity to adapt, it creates a mismatch that heightens injury risk. Gabbett highlights the "training-injury prevention paradox," where insufficient training leaves athletes underprepared, while excessive load without recovery increases susceptibility to acute and overuse injuries [28]. Fatigue exacerbates this mismatch by impairing proprioception and motor control, often leading to biomechanical compensations that overload tissues [6].

Psychological and Cognitive Influences

Psychological readiness and decision-making during play also influence injury mechanisms. Fear of reinjury, anxiety, or lack of focus can alter movement patterns, increasing the likelihood of errors that lead to injury. For example, an athlete recovering from an anterior cruciate ligament (ACL) reconstruction may subconsciously adopt a more cautious gait, inadvertently placing additional stress on other joints or muscle groups [37].

Multifactorial nature of injury patterns

**Figure 2:**
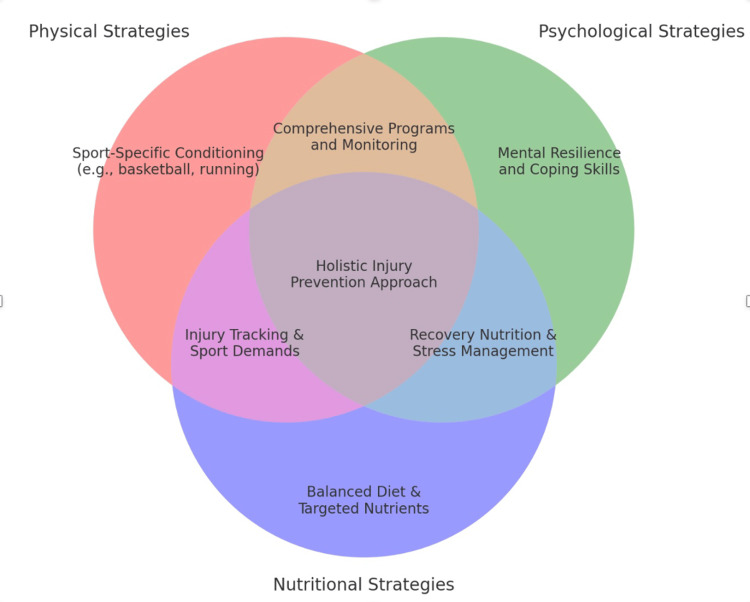
Holistic injury prevention approach in sports

Bittencourt et al.'s complex systems approach underscores that injuries are not merely the sum of isolated risk factors but emerge from their dynamic interactions. Identifying isolated predictors like poor biomechanics or fatigue is insufficient without understanding how these factors converge under specific circumstances, such as during high-stress competition or on unfamiliar surfaces [27]. This holistic perspective calls for integrative analyses that account for the interplay of physical, psychological, and environmental elements in shaping injury risk (Figure 2).

Sport Activity Analysis

Sport activity analysis is a vital tool for understanding the mechanical loads and stresses imposed on athletes during their activities, providing the foundation for effective injury prevention. This approach combines biomechanical insights, workload management, and sport-specific training adaptations to minimize injury risks and optimize performance. By addressing the physical demands of different sports, tailored interventions can prevent both acute and overuse injuries (Table 3).

**Table 3:** Key components and applications of sport activity analysis GPS: Global Positioning System; FIFA 11+: Federation of International Football Association 11+

Aspect of analysis	Purpose	Examples
Biomechanical assessments	Identify movement patterns or inefficiencies that increase injury risk	Motion capture systems to analyze running gait, force plates to assess landing mechanics
Workload monitoring	Track training intensity and recovery to prevent overtraining and fatigue-related injuries	Use of GPS trackers and heart rate monitors to balance workload and recovery
Tailored conditioning programs	Enhance muscular resilience and adapt to sport-specific physical demands	Programs like FIFA 11+ program for soccer players focusing on strength, balance, and agility
Sport-specific training	Reduce risks associated with repetitive movements unique to each sport	Plyometric exercises for basketball players, shoulder stabilization drills for swimmers
Gradual return-to-play protocols	Ensure a safe and progressive transition back to competition while minimizing reinjury risk	"Control to Chaos" continuum guiding structured progression from controlled exercises to dynamic competition

Biomechanical insights and injury prevention: Biomechanical assessments are essential for understanding movement patterns that may increase an athlete's risk of injury. Many sports, particularly those involving repetitive or high-impact motions, impose specific stresses on the body. For instance, activities that require jumping and landing, such as basketball or volleyball, place significant strain on lower body joints, potentially leading to injuries such as ligament sprains or tears [38]. Similarly, endurance sports like running often expose athletes to repetitive strain on their muscles and bones, contributing to overuse injuries such as stress fractures [33].

By leveraging advanced technologies, such as motion capture systems and force plates, practitioners can identify inefficiencies or abnormalities in an athlete's movement. Issues like improper alignment, muscle imbalances, or joint instability can significantly increase the risk of injury. For example, poor pelvic positioning during dynamic activities can compromise stability and elevate the likelihood of strains or tears [29].

Corrective measures, such as neuromuscular training, targeted strength conditioning, and flexibility exercises, are effective in addressing these biomechanical issues. These strategies not only reduce injury risks but also enhance performance by improving movement efficiency and resilience under the physical demands of sport [24].

Workload management: Effective workload management is crucial for preventing overuse injuries. Overtraining, characterized by excessive physical stress without adequate recovery, is a known risk factor for injury [28]. Wearable technologies, such as Global Positioning System (GPS) trackers and heart rate monitors, allow precise monitoring of training loads, enabling coaches to balance workload and recovery periods. For instance, reducing high-speed running efforts in soccer players during intense training phases has been shown to decrease hamstring strain risk [20].

Tailored conditioning programs: Tailored conditioning programs enhance muscular resilience and adapt to the specific demands of a sport. Programs such as the Federation of International Football Association 11+ (FIFA 11+) incorporate strength, balance, and agility exercises designed to reduce the incidence of injuries in soccer players. Research demonstrates that teams implementing the FIFA 11+ program experience significantly fewer injuries than those that do not [39].

Conditioning should also address sport-specific demands. For instance, basketball players benefit from plyometric exercises that mimic explosive movements, while swimmers require targeted shoulder stabilization routines to prevent overuse injuries from repetitive overhead strokes. These interventions reduce injury risks while improving functional performance.

Gradual return-to-play protocols: Reintegrating athletes into competition requires a structured, progressive approach to minimize reinjury risk. The "Control to Chaos" continuum, a framework for rehabilitation, guides athletes through controlled exercises to more dynamic and unpredictable movements, replicating real-game scenarios [26]. This ensures that athletes are not only physically ready but also psychologically prepared to handle the stresses of competition.

The Psychological Dimension of Injury Prevention

Psychological resilience plays a critical role in preventing injuries and aiding recovery. Injuries are not only physical setbacks but also psychological challenges that can disrupt an athlete's confidence, motivation, and mental well-being. Sport activity analysis, when integrated with psychological strategies, ensures a more holistic approach to injury prevention.

Reinjury anxiety and mental resilience: Reinjury anxiety is common among athletes returning to competition after an injury. Studies show that this anxiety often leads to cautious or altered movements, increasing the risk of subsequent injuries [40]. Psychological interventions, such as visualization, goal setting, and self-talk, help athletes rebuild confidence. These techniques have been shown to reduce anxiety and improve focus, enabling a smoother return to play [37].

Mental training for injury prevention: Prevention programs incorporating psychological training can prepare athletes to cope with the pressures of competition. Goal setting, imagery, and mindfulness techniques enhance focus and stress management, reducing distractions that often lead to injuries [41]. Mindfulness-based practices, in particular, improve an athlete's body awareness, helping them recognize and adjust to physical strain before it becomes injurious [42].

Psychological support during rehabilitation: During rehabilitation, maintaining mental engagement is crucial. Injured athletes often experience feelings of frustration, depression, or isolation, which can hinder recovery. Providing psychological support through structured counseling, group therapy, or peer mentorship programs improves emotional resilience and adherence to rehabilitation protocols [14]. Research indicates that athletes who actively participate in psychological programs recover faster and have a lower risk of reinjury. 

Sleep during rehabilitation: Sleep plays a critical role in injury prevention by supporting recovery, maintaining physical performance, and optimizing neuromuscular function. Adequate sleep enhances tissue repair, reduces inflammation, and helps maintain hormonal balance, all of which are essential for reducing injury risk. Moreover, insufficient sleep has been associated with impaired reaction times, diminished proprioception, and reduced cognitive function, which can increase the likelihood of accidents or biomechanical compensations leading to overuse injuries. Research suggests that athletes who consistently get less than seven hours of sleep per night are significantly more likely to sustain injuries compared to those who sleep longer. Additionally, sleep quality influences muscle strength and endurance, further emphasizing its importance in athletic performance and injury prevention [43,44].

Reducing fear-avoidance behaviors:Fear-avoidance behaviors, where athletes consciously or unconsciously avoid movements associated with prior injuries, can lead to maladaptive patterns and increased strain on other body parts. Psychological interventions aimed at overcoming these behaviors include graduated exposure therapy and cognitive restructuring, which help athletes regain trust in their physical capabilities [13].

The Role of Nutrition in Injury Prevention

Nutrition is a cornerstone of sports injury prevention, playing a pivotal role in enhancing resilience, recovery, and long-term athlete performance (Table 4). The integration of strategic nutritional practices directly addresses key physiological factors that contribute to injury risk. Macronutrients are foundational to this strategy: carbohydrates replenish glycogen stores, ensuring sustained energy and preventing fatigue, a common precursor to form breakdown and injury in endurance sports like running. The brain's reliance on glucose highlights the importance of recommending carbohydrate intake during physical activity. Current guidelines suggest consuming approximately 40-90 grams of carbohydrates per hour of effort [45,46]. Proteins are vital for muscle repair and adaptation, particularly after high-intensity training or competition. Research indicates that consuming 20-30 grams of protein shortly after exercise optimizes muscle protein synthesis, aiding in tissue repair and reducing the risk of overuse injuries (Smith et al., 2011). Healthy fats, especially omega-3 fatty acids, mitigate inflammation, promoting joint health and reducing the chronic inflammation associated with repetitive stress activities such as swimming or tennis [47].

**Table 4:** Key nutritional strategies for injury prevention and recovery in athletes IU: international unit

Nutritional element	Role in injury prevention	Examples
Carbohydrates	Prevents muscle/mental fatigue and supports energy for sustained performance and recovery	Consume as part of balanced meals, especially around training sessions
Proteins	Essential for muscle repair and adaptation, reducing the risk of overuse injuries	Ingest 20-30 grams post-training for muscle recovery; adjust intake based on activity level
Healthy fats (e.g., omega-3 fatty acids)	Supports joint health and reduces chronic inflammation linked to repetitive stress	Include foods like fish, walnuts, and flaxseeds regularly in the diet, especially in quality oils (camelina, rapeseed, flax, etc.)
Calcium and vitamin D	Strengthens bones and reduces the risk of stress fractures, particularly in high-impact sports	Supplement with 800 IU/day of vitamin D and 2,000 mg of calcium; focus on dairy and leafy greens
Antioxidants	Protects muscles from oxidative stress, speeding up recovery and reducing soreness	Incorporate berries, citrus fruits, and leafy greens for their antioxidant content
Hydration	Maintains joint lubrication, muscle elasticity, and optimal coordination to prevent injuries	Ensure consistent hydration, particularly before, during, and after high-impact activities
Anti-inflammatory nutrients	Reduces chronic inflammation and supports tissue resilience against repetitive strain	Use turmeric, ginger, and omega-3 supplements to mitigate inflammation
Timing of nutrient intake	Enhances recovery by promoting muscle repair and glycogen replenishment post-training	Consume protein and carbohydrates within 30 minutes postexercise for optimal recovery

Micronutrients also play an essential role in structural integrity and recovery. Calcium and vitamin D are critical for bone health, with deficiencies linked to increased risks of stress fractures, particularly in athletes engaged in high-impact sports like basketball and gymnastics. Supplementation with 800 IU/day of vitamin D and 2,000 mg of calcium has been shown to significantly reduce these risks [48]. Antioxidants such as vitamins C and E protect muscle tissue from oxidative stress, reducing soreness and enhancing recovery, particularly after prolonged or intense training sessions [49].

Hydration is a crucial but often overlooked component of injury prevention. Proper hydration maintains joint lubrication, muscle elasticity, and neuromuscular coordination. Dehydration not only increases the risk of cramps and soft tissue injuries but also impairs thermoregulation, exacerbating fatigue-related performance issues [47]. Anti-inflammatory nutrients such as turmeric and ginger, combined with collagen and vitamin C, further support connective tissue health. These nutrients enhance tendon and ligament resilience, critical for athletes in sports with high rotational demands like soccer or gymnastics [50].

The timing of nutrient intake is equally important. Postexercise consumption of carbohydrates and proteins within 30 minutes replenishes glycogen stores and supports muscle repair, a strategy that significantly reduces recovery time and injury risk [45]. Integrating anti-inflammatory foods into recovery meals helps manage inflammation and prepares the body for subsequent training demands. Personalized nutrition plans, tailored to an athlete’s sport-specific and individual needs, provide targeted benefits. For instance, endurance athletes may prioritize carbohydrate loading, while strength athletes may require higher protein intakes to support muscle development.

Incorporating these nutritional strategies into a comprehensive injury prevention framework amplifies their efficacy. When combined with physical training, workload monitoring, and psychological resilience practices, nutrition forms a holistic approach that addresses both the physical and systemic factors contributing to injuries. By adopting evidence-based nutritional practices, athletes not only minimize injury risks but also enhance their capacity for recovery, ensuring sustained performance and career longevity.

## Conclusions

The prevention of sports injuries, particularly recurrent injuries, requires a comprehensive, multifactorial approach that integrates physical, psychological, and nutritional strategies. This article highlights the critical interplay between biomechanical insights, workload management, psychological resilience, and optimal nutrition in mitigating injury risks and enhancing athletic performance. Biomechanical assessments remain a cornerstone of injury prevention, enabling the identification and correction of movement inefficiencies that predispose athletes to harm. When combined with tailored strength and conditioning programs, these interventions strengthen musculoskeletal resilience and enhance functional performance. Concurrently, effective workload management, facilitated by wearable technologies and structured training plans, ensures that athletes maintain a balance between training intensity and recovery, reducing the risk of both acute and overuse injuries. The psychological dimension of injury prevention is equally vital. Reinjury anxiety, fear-avoidance behaviors, and mental disengagement can all compromise recovery and increase susceptibility to new injuries. Psychological interventions, such as mindfulness, goal setting, and cognitive restructuring, help athletes overcome these barriers, fostering confidence and readiness to perform at their best. Nutrition, too, plays a pivotal role in sustaining an athlete's resilience and recovery. Balanced macronutrient intake supports energy availability and tissue repair, while micronutrients like calcium, vitamin D, and antioxidants contribute to bone health and reduce inflammation. The timing and personalization of nutrient intake further amplify these benefits, ensuring athletes meet the specific demands of their sport. Ultimately, injury prevention is not about addressing isolated factors but about adopting an integrated, individualized approach that considers the unique needs of each athlete. By bridging physical, psychological, and nutritional dimensions, this holistic framework not only reduces injury risks but also empowers athletes to sustain peak performance and prolong their careers, ensuring they remain competitive and resilient in the demanding world of sports.
